# Gastrointestinal Involvement in SARS-CoV-2 Infection

**DOI:** 10.3390/v14061188

**Published:** 2022-05-30

**Authors:** Tsung-Hsien Chen, Ming-Tse Hsu, Ming-Yang Lee, Chu-Kuang Chou

**Affiliations:** 1 Department of Internal Medicine, Ditmanson Medical Foundation Chia-Yi Christian Hospital, Chiayi 60002, Taiwan; cych13794@gmail.com; 2 Division of Gastroenterology and Hepatology, Department of Internal Medicine, Ditmanson Medical Foundation Chia-Yi Christian Hospital, Chiayi 60002, Taiwan; hsub1686@gmail.com; 3 Division of Hemato-Oncology, Department of Internal Medicine, Ditmanson Medical Foundation Chia-Yi Christian Hospital, Chiayi 60002, Taiwan; 4 Min-Hwei Junior College of Health Care Management, Tainan 73658, Taiwan; 5 Obesity Center, Ditmanson Medical Foundation Chia-Yi Christian Hospital, Chiayi 60002, Taiwan; 6 Clinical Trial Center, Ditmanson Medical Foundation Chia-Yi Christian Hospital, Chiayi 60002, Taiwan

**Keywords:** SARS-CoV-2, gastrointestinal involvement, prevention, oral vaccine, microbiota

## Abstract

SARS-CoV-2 has evolved into a virus that primarily results in mild or asymptomatic disease, making its transmission more challenging to control. In addition to the respiratory tract, SARS-CoV-2 also infects the digestive tract. Some gastrointestinal symptoms occur with or before respiratory symptoms in patients with COVID-19. Respiratory infections are known to cause intestinal immune impairment and gastrointestinal symptoms. When the intestine is inflamed, cytokines affect the lung immune response and inflammation through blood circulation. The gastrointestinal microbiome may be a modifiable factor in determining the risk of SARS-CoV-2 infection and disease severity. The development of oral SARS-CoV-2 vaccine candidates and the maintenance of gut microbiota profiles may contribute to the early control of COVID-19 outbreaks. To this end, this review summarizes information on the gastrointestinal complications caused by SARS-CoV-2, SARS-CoV-2 infection, the gastrointestinal–lung axis immune response, potential control strategies for oral vaccine candidates and maintaining intestinal microbiota homeostasis.

## 1. Introduction

A high-incidence respiratory illness, coronavirus disease 2019 (COVID-19), caused by the human-to-human transmission of the novel coronavirus (severe acute respiratory syndrome–coronavirus 2 (SARS-CoV-2)) was identified in December 2019 [1]. COVID-19 has since become a global pandemic [2], leading the World Health Organization to declare a global public health emergency [3]. SARS-CoV-2 is more transmissible than SARS-CoV [4]. Different variants of SARS-CoV-2 have been reported, such as Alpha (B.1.1.7), Beta (B.1.351), Gamma (P.1), Delta (B.1.617.2), and Omicron (B.1.1.529). Delta variants (B.1.617.2) with increased infectivity, severe disease course, and reduced therapeutic efficacy were designated as variants of concern (VOC) on 15 June 2021 [5]. However, on 14 April 2022, the U.S. government SARS-CoV-2 Interagency Group based on a significant and sustained reduction in its national and regional proportions over time, stated that Delta (B.1.617.2) did not currently pose a significant risk to public health in the United States, thereby downgrading Delta from VOC to variant being monitored [5]. Additionally, the Omicron variant (B.1.1.529), a new severely mutated SARS-CoV-2 variant, was designated as a VOC on 30 November 2021, based on the following: detection of cases attributed to Omicron in multiple countries, the number and locations of substitutions in the spike protein, reduction in neutralization by sera from vaccinated or convalescent individuals, and reduced susceptibility to certain monoclonal antibody treatments [5,6].

**Figure 1:**
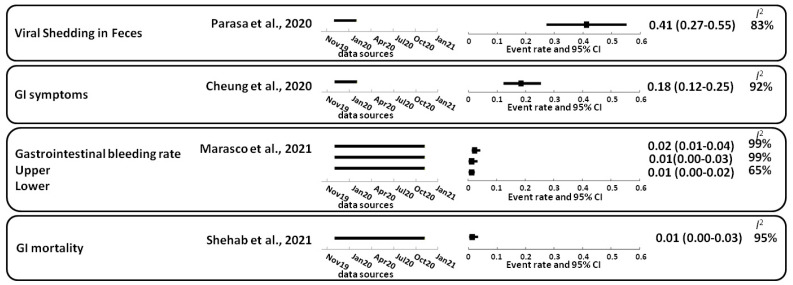
Gastrointestinal symptoms, bleeding, and mortality in patients with COVID-19. The 95% confidence interval (95% CI); random-effects model estimate. For the *I*^2^ statistic, the level of heterogeneity was defined as low (25–50%), moderate (50–75%), or high (>75%). Squares indicate proportions. GI, gastrointestinal. References: Cheung et al., 2020 [9]; Marasco et al., 2021 [11]; Parasa et al., 2020 [7]; Shehab et al., 2021 [12].

SARS-CoV-2 primarily attacks host lung cells. Thus, the main symptom of COVID-19 is a respiratory infection. SARS-CoV-2 RNA can be detected in the stool in approximately 41% (27–55%) of COVID-19 cases. Viral shedding in feces was 41% (27–55%) [7], accompanied by gastrointestinal symptoms in approximately 18% (12–25%), such as nausea, vomiting, and diarrhea [8,9]. SARS-CoV-2 virions can be removed via mucociliary clearance or enter the gastrointestinal tract from the esophagus. While gastrointestinal symptoms are common, cases of COVID-19 with gastrointestinal symptoms are more likely to develop acute respiratory distress and liver damage and have a poorer prognosis [10]. For instance, the gastrointestinal bleeding rate was 2% (1–4%) [11], and gastrointestinal mortality was 1% (0–3%) [12] (Figure 1). Therefore, more attention has been paid recently to the gastrointestinal manifestations of SARS-CoV-2 [13,14,15,16,17].

## 2. Gastrointestinal Complications Caused by SARS-CoV-2

Fever and respiratory symptoms are commonly present in patients with COVID-19; however, digestive symptoms, including anorexia, nausea, vomiting, and diarrhea are also commonly reported [9,13,14,15,16,17]. Gastrointestinal imaging findings include bowel wall thickening, sometimes with hyperemia and mesenteric thickening, fluid-filled large bowel, and rarely pneumatosis and ischemia [18].

**Figure 2:**
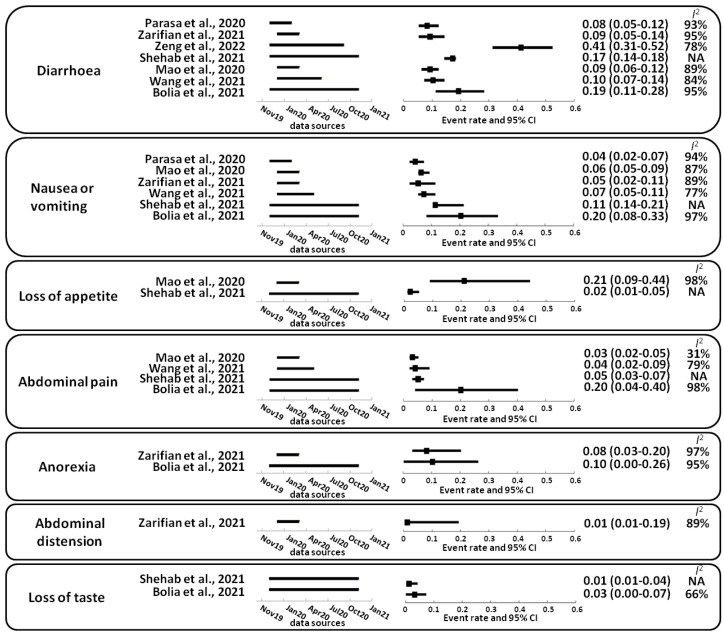
Gastrointestinal symptoms described in patients with COVID-19. The 95% confidence interval (95% CI); random-effects model estimate. For the *I*^2^ statistic, the level of heterogeneity was defined as low (25–50%), moderate (50–75%), or high (>75%). Squares indicate proportions. NA, not available. References: Bolia et al., 2021 [23]; Mao et al., 2020 [21]; Parasa et al., 2020 [7]; Shehab et al., 2021 [12]; Wang et al., 2021 [30]; Zarifian et al., 2021 [19]; Zeng et al., 2022 [20].

In patients with COVID-19, the following prevalence of gastrointestinal symptoms has been reported in systematic reviews and meta-analysis: diarrhea (8–17%) [7,12,19,20,21,22,23], nausea or vomiting (4–20%) [7,12,19,21,22,23], loss of appetite (2–21%) [12,21], abdominal pain (3–20%) [12,21,22,23], anorexia (8–10%) [19,23], abdominal distension (1%) [19] and loss of taste (1–3%) [12,23] (Figure 2). Infectious diarrhea and malabsorption caused by SARS-CoV-2 infection may be due to the dysregulation of intestinal ion transporters [24], leading to inflammation and gastrointestinal symptoms [25]. Most gastrointestinal symptoms associated with COVID-19 are mild [26]. Diarrhea caused by SARS-CoV-2 may be the first symptom in patients with COVID-19 [21]. Additionally, a subset of patients with COVID-19 develop isolated gastrointestinal symptoms that may precede the development of respiratory symptoms [27,28] or have only digestive symptoms throughout the disease (2.9–16%) [13,21,27,28,29].

The incidence of gastrointestinal manifestations was higher in the later period of the pandemic than in the early period [31]. Patients with gastrointestinal symptoms are at an increased risk of developing acute respiratory distress syndrome [21,32]. The proportion of patients with severe COVID-19 and critically ill patients was significantly higher in those with gastrointestinal symptoms [21,31]. Patients with severe COVID-19 have a higher abdominal pain incidence than patients with non-severe disease [21,31]. There were no significant differences in loss of appetite, diarrhea, nausea, or vomiting in severely and non-severely ill patients with COVID-19 [21,30]. Significantly lower mortality in patients with COVID-19 with gastrointestinal symptoms showed better clinical outcomes than in patients without gastrointestinal symptoms [33,34,35]. 

COVID-19 exposure may increase the risk of thromboembolic events and associated ischemia [36,37,38], including limb venous thrombosis and pulmonary embolism [36,39,40,41]. Thromboembolic events in the gastrointestinal system, including mesenteric ischemia, are a potentially fatal clinical emergency with high mortality [42]. Large vessel arterial/venous thrombosis can be present in almost half of the patients with intestinal ischemia in COVID-19. The overall mortality of these patients with gastrointestinal ischemia and radiographically diagnosed mesenteric ischemia is 38.7 and 40%, respectively [43]. COVID-19 is known to be associated with elevated transaminase levels, as well as higher rates of intestinal obstruction and intestinal ischemia [32].

## 3. SARS-CoV-2 Infection

Coronaviruses are enveloped, positive-sense RNA viruses containing an ssRNA genome with a 5′-terminal cap and 3′-polyadenylation that infect various mammalian and avian species [44]. SARS-CoV-2 virus particles are formed from an envelope and membrane alone, with a spike protein forming the viral envelope. The spike protein enables the virus to attach the host cell membrane, and the nucleocapsid protein holds the virus’s RNA genome [45]. When the coronavirus genome is released into the host cytoplasm, a complex and highly regulated viral gene expression program is triggered [46]. Through co-translational and post-translational mechanisms, the viral proteases nonstructural proteins (nsp) 3, 5, and 16 are processed and released from pp1a (nsp1–11) and pp1ab (nsp1–10, nsp12–16). Fifteen of these constitute the viral replication and transcription complex [47], including RNA processing and modification enzymes, and RNA proofreading functions. The SARS-CoV-2 infection triggers an inflammatory immune response, during which lymph node-derived helper T cells and cytotoxic T cells infiltrate the site of infection to eliminate virus-infected cells [48].

### 3.1. Initial Steps of SARS-CoV-2 Infection

**Figure 3:**
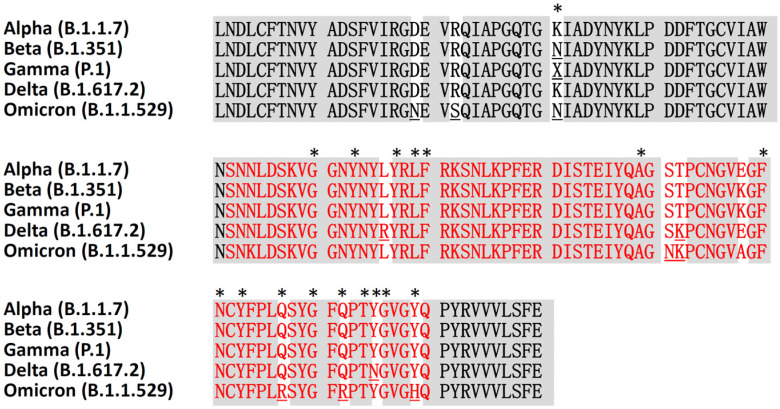
Multiple sequence alignment of the receptor-binding domains amino acid sequences of five SARS-CoV-2 variants. Contacting residues in the SARS-CoV-2 receptor-binding domain are indicated by an asterisk. The receptor-binding motif sequence is shown in red. The surface glycoprotein receptor-binding domain sequences are from GenBank, with the following accession protein ID: UFZ12739.1 (Alpha), QWW93436.1 (Beta), QWW27582.1 (Gamma), UAL04647.1 (Delta), and UKO09871.1 (Omicron).

Virus entry into host cells is an essential part of the infection. SARS-CoV-2 enters and regulates cellular factors to promote replication [49], and infects cells through the endocytosis mechanism or fusion of the viral envelope with the host cell plasma membrane [46]. Coronaviruses encode surface glycosylated transmembrane proteins and spike protein-containing receptor-binding domains and fusion domains to mediate virus entry [46,50]. Spike protein subunits S1 and S2 mediate the attachment, with the simultaneous binding of two S-glycoprotein trimers to the cell surface protein angiotensin-converting enzyme 2 (ACE2) [51,52]. In addition, the cellular transmembrane protease serine 2 (TMPRSS2), which has a serine protease activity, is also required to initiate the spike protein priming [53]. The alignment of receptor-binding domain sequences of SARS-CoV-2 variants revealed that the Omicron variant has multiple mutations in the receptor-binding motif (Figure 3). Six mutations in the spike protein receptor-binding domain of the Omicron variant (B.1.1.529), including S477N, T478K, E484A, Q493R, Q498R, and Y505H, are responsible for the higher affinity for ACE2 (Figure 3). In addition, a mutation position G496S was also found in other Pango lineages of Omicron. Recent molecular dynamics simulations and ELISA bioassay results show that the Omicron variant binds human ACE2 with comparable binding affinity to wild-type SARS-CoV-2, but much weaker than the Delta variant [54]. However, the Omicron variant has a high risk of immune evasion and thus potential reduction in neutralization by postvaccination sera may make it easy to spread [54].

### 3.2. Angiotensin-Converting Enzyme 2 Expression and Genetic Variation

**Figure 4:**
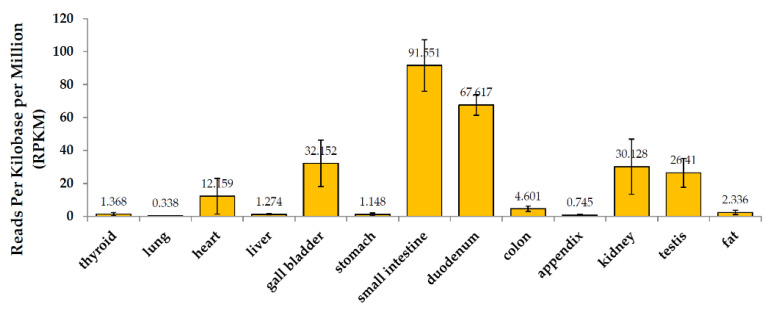
mRNA expression of ACE2 in healthy human tissues. Source: BioProject: PRJEB4337.

The expression and distribution of ACE2 in humans is a potential infection route of SARS-CoV-2. The binding affinity of ACE2 to the SARS-CoV-2 outer domain is about 10- to 20-fold higher than that of SARS-CoV [52]. Transmembrane ACE2 is highly expressed in the ciliated, goblet, and surfactant-producing type II alveolar cells and type II epithelial cells [55]. These cells are mainly located in the lung, intestine (small intestinal epithelium), esophagus and pancreas, heart, kidney, and liver. However, lung ACE2 expression was concentrated in a small population of type II alveolar cells, likely resulting in relatively low lung ACE2 expression in the analysis of BioProject (PRJEB4337, Figure 4) [56,57]. Additionally, the gastrointestinal tract may be susceptible to SARS-CoV-2 infection due to widely expressed ACE2 and TMPRSS2 in the intestine, causing direct damage [14,58,59,60,61,62]. In children infected with SARS-CoV-2, the manifestations of common intestinal symptoms may be related to the higher expression of intestinal ACE2 in children [63].

Furthermore, amino acids regulate the secretion of antimicrobial peptides to maintain intestinal microbiota homeostasis [64]. ACE2 plays a vital role in the expression of amino acid transporters in the small intestine. ACE2 regulates amino acid uptake in intestinal epithelial cells, the expression of antimicrobial peptides, and gut microbiome ecology [65,66]. ACE2 can absorb nutrients from digested food and maintain osmotic and electrolyte balance throughout the gastrointestinal epithelial cells by regulating sodium-dependent amino acid and glucose transporters in the brush border of enterocytes [66]. ACE2 is also a key enzyme in the renin–angiotensin system (RAS) [67] and plays an important role in regulating intestinal inflammation and diarrhea [66]. Thus, the interaction between SARS-CoV-2 and ACE2 may disrupt the function of ACE2 and cause diarrhea.

Recent studies have found that genetic components of the ACE2 and TMPRSS2 genes can mediate the effects on the severity of COVID-19. East Asian populations have much higher allele frequencies in ACE2 expression quantitative trait locus variants, which may contribute to the differential susceptibility or response to SARS-CoV-2 [68]. The frequency of TMPRSS2 upregulated variants was higher in European and American populations than in Asian populations, implying that European and American populations may be relatively susceptible to SARS-CoV-2 infection [69]. A study of the genetic component of COVID-19 severity in Italians reported that ACE2 was not associated with COVID-19 severity/sex bias, but TMPRSS2 levels and genetic variation may be associated with higher susceptibility to COVID-19 severity [70].

### 3.3. SARS-CoV-2 Infection in the Gastrointestinal Tract

Viruses in the gastrointestinal tract can contribute to host health or disease by interacting with the mucus layer, epithelial cells, and potentially lamina propria immune cells. Variation in the gut virome may contribute to phenotypic variation by modulating the immunophenotype rather than acting as a pathogen [71]. SARS-CoV-2 RNA was detected in the stool of patients with COVID-19, implying that SARS-CoV-2 may be transmitted through the fecal–oral route [8,72,73]. A large proportion (29–53.4%) of patients with COVID-19 tested positive for SARS-CoV-2 RNA in stool [73,74]. An endoscopic sampling of different parts of the patient’s gastrointestinal tract was performed, and viral RNA was also detected in the esophagus, stomach, duodenum, and rectum [74]. Additionally, the SARS-CoV-2 viral nucleocapsid protein was detected in the cytoplasm of gastric, duodenal, and rectal glandular epithelial cells [59]. However, it is unclear whether the virus in the digestive system is derived from cellular debris from the respiratory system or consists of replicas in the digestive tract [75]. Therefore, early measures should be taken to prevent fecal–oral transmission [75].

The gastrointestinal tract is confirmed as an alternative route for SARS-CoV-2 infection in rhesus monkeys [76]. The SARS-CoV-2 virus can infect and replicate in human intestinal tissue [61], and viral toxin-mediated cellular damage causes gastroenteritis-like symptoms including diarrhea, nausea, vomiting, and abdominal pain. Fecal PCR returns a positive result in 36–53% of patients with COVID-19, approximately 2–5 days later than a sputum PCR positive [31]. Respiratory samples from patients with COVID-19 were positive for SARS-CoV-2 RNA for 16.7 days, but their stool samples were positive for 27.9 to 47 days [21,77]. Notably, the intragastric inoculation of rhesus monkeys with SARS-CoV-2 leads to dysfunctions in both respiratory and digestive systems [76]. Inflammatory cytokines are speculated to be a possible link in the pathogenesis of SARS-CoV-2 between the respiratory and digestive systems [76].

### 3.4. SARS-CoV-2 and Gut Microbiome

In a healthy gastrointestinal tract, the microbiota is rich in beneficial bacteria that help to maintain intestinal homeostasis, promote protective intestinal immune responses at mucosal surfaces, and limit excessive mucosal inflammation [78]. It consists of more than 100 trillion microorganisms and thousands of bacterial species [79,80]. The microbiota maintains a symbiotic relationship with the gut environment and forms a mutually beneficial relationship with the host. The gut microbiota can influence the maturation, development, and function of immune cells, as well as the activation of peripheral immune cells, including cellular and humoral immunity. Innate and adaptive immune cells are activated by the disruption of gut barrier integrity and release pro-inflammatory cytokines into the circulatory system, leading to systemic inflammation [81]. The entry of inflammatory cells, including neutrophils and lymphocytes, into the intestinal mucosa disrupts the gut microbiota [82].

Viral infection alters the permeability of the intestinal wall, leading to malabsorption by enterocytes [64]. Fecal calprotectin levels were elevated in patients infected with SARS-CoV-2, confirming that SARS-CoV-2 causes intestinal inflammation [83]. A recent comprehensive systematic review confirmed that the most common alteration in the bacterial composition of patients with COVID-19 was a depletion in the genera *Ruminococcus, Alistipes*, *Eubacterium*, *Bifidobacterium*, *Faecalibacterium*, *Roseburia*, *Fusicathenibacter*, and *Blautia*, as well as the enrichment of *Eggerthella*, *Bacteroides*, *Actinomyces*, *Clostridium*, *Streptococcus*, *Rothia*, and *Collinsella* [84]. Changes to the gut microbiome composition and function affect the respiratory tract through the common mucosal immune system, and respiratory dysbiosis also affects the digestive tract through immune regulation [85].

Alterations in the gut microbiome are associated with severity and poor prognosis in patients with COVID-19, such as increases in *Bacteroides*, *Parabacteria*, *Clostridium*, *Bifidobacterium*, *Ruminococcus*, *Campylobacter*, *Rotella*, *Corynebacterium Pseudomonas*, *Megacoccus*, *Enterococcus*, and *Aspergillus*, as well as reductions in *Roseburia*, *Eubacterium*, *Lachnospira*, *Faecalibacterium*, and *Firmicutes/Bacteroidetes* ratios [84]. The fungal gut microbiota of patients with severe/critical COVID-19 was characterized by decreased diversity, richness, and evenness, and increased relative abundance of *Ascomycota phylum* compared with non-severe COVID-19 [86]. Patients with severe SARS-CoV-2 infection had significantly lower bacterial diversity, and lower relative abundances of *Bifidobacterium*, *Faecalibacterium*, and *Roseobacter* in the gut microbiome, as well as increased *Bacteroides* spp. [87]. Thus, during the SARS-CoV-2 pandemic, gut microbiota correction may help to improve population immunity and protect public health [88].

## 4. Host Immune Response Induced by SARS-CoV-2

During the early stages of SARS-CoV infection, dendritic cells and macrophages exhibit a delayed release of cytokines and chemokines, followed by low concentrations of antiviral interferons and high concentrations of proinflammatory cytokines and chemokines [89,90,91]. Rapidly elevated cytokines and chemokines attract large numbers of inflammatory cells such as neutrophils and monocytes, causing tissue damage. Increased relative frequencies of circulating activated CD4+ and CD8+ T cells and plasmablasts are present in patients with COVID-19 [92]. Lymphocytosis is a common feature in patients with severe COVID-19 infection, with markedly reduced numbers of CD4+ T cells, CD8+ T cells, B cells, and natural killer cells [93].

**Figure 5:**
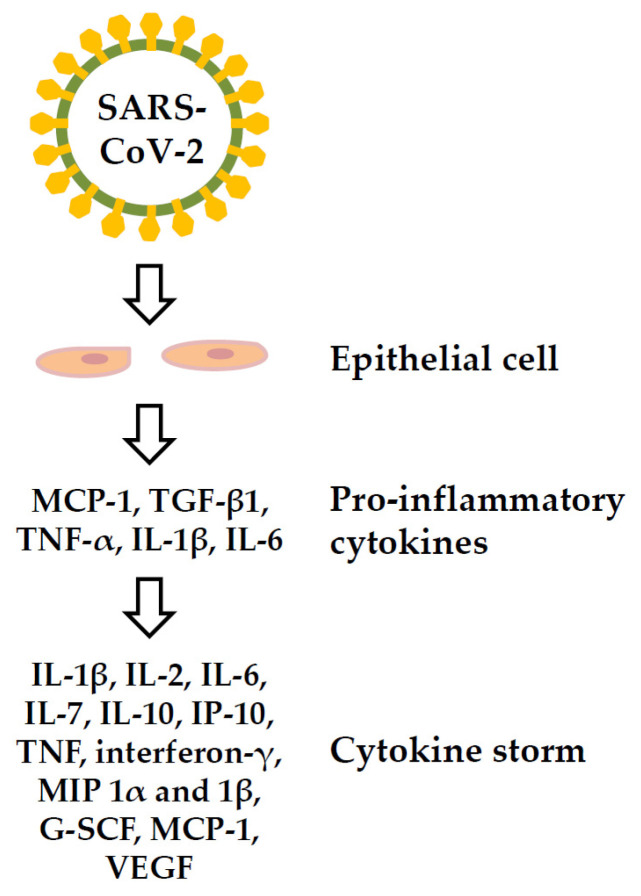
Cytokine storm of the COVID-19 disease. G-SCF: plasma granulocyte colony-stimulating factor, IL: interleukin; IP: interferon-inducible protein; MCP-1: monocyte chemokine-1, MIP: macrophage inflammatory protein, TGF-β1: tumor growth factor-β1, TNF: tumor necrosis factor, VEGF: vascular endothelial growth factor.

ACE2-expressing cells in patients with COVID-19 express pro-inflammatory cytokines (PICs) including monocyte chemokine-1 (MCP-1), tumor growth factor-β1 (TGF-β1), tumor necrosis factor (TNF)-α, interleukin (IL)-1β, and IL-6 [94]; these cytokines can cause cytokine storms and lead to multiple organ damage. Cytokine storms may contribute to the pathogenesis of COVID-19, and may directly lead to immune cell death [95,96]. The serum cytokines showing elevated levels in patients with COVID-19-related cytokine storms include IL-1β, IL-2, IL-6, IL-7, IL-10, interferon-inducible protein (IP)-10, TNF, interferon-γ, macrophage inflammatory protein (MIP) 1α and 1β, plasma granulocyte colony-stimulating factor (G-SCF), MCP-1, and vascular endothelial growth factor (VEGF) [97,98] (Figure 5). High levels of IL-6 were associated with reduced survival in patients with COVID-19 [99].

### 4.1. Immune Response in the Gut Affects Gastrointestinal Tract

Digestive symptoms associated with SARS-CoV-2 infection may result from direct viral attack as well as tissue and organ damage from the immune response [59,100]. SARS-CoV-2 infection induces early neutralizing antibody responses, including systemic IgA and a peripheral expansion of IgA plasmablasts with mucosal homing potential and systemic IgG [101,102]. IL1β, IL4, IL5, IL6, G-CSF, granulocyte-macrophage colony-stimulating factor (GM-CSF), interferon-γ, IL2, IL10, I-12/23, IL13, IL15, IL17A, MCP-1, MIP-1β, MIP-1α, sCD40L, TGFα, TNFα, VEGF A, and IL18 were upregulated in the digestive tissues of rhesus monkeys after SARS-CoV-2 infection [76]. Inflammatory cytokines are induced in more segments of the gastrointestinal tract as the SARS-CoV-2 infection progresses. Subsequently, anti-inflammatory or protective cytokines such as G-CSF, IL4, IL6, IL13, IL18, MIP-1β, and TNFα are increasingly expressed in the gastrointestinal segment [76].

**Figure 6:**
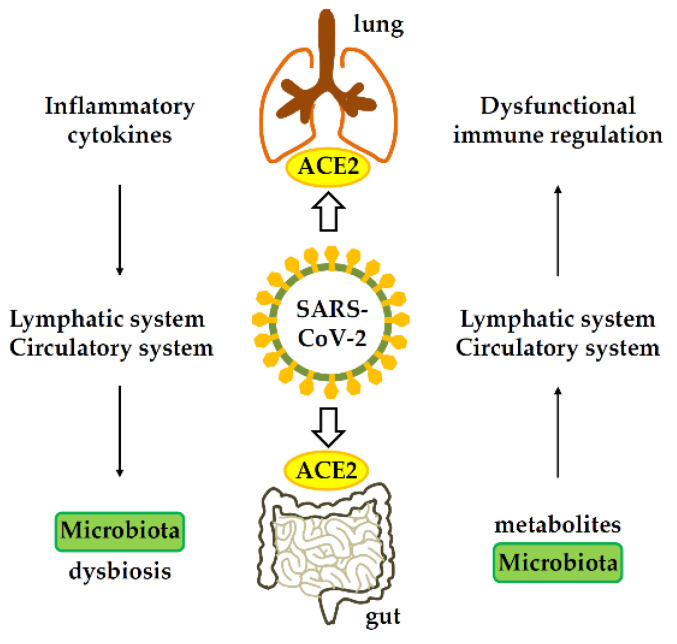
Gastrointestinal-lung axis in COVID-19. ACE2, angiotensin-converting enzyme 2.

Pulmonary-derived CC chemokine receptor 9 (CCR9)^+^ CD4^+^ T cells are increased after viral infection [103]. Effector CD4^+^ T cells are critical for the development of intestinal mucosal immunity and chronic enteritis, and CCR9 is a chemokine receptor necessary for CD4^+^ T cell entry into the small intestine [103]. When the intestinal epithelium expresses the C-C motif chemokine ligand 25 (CCL25) [104], it can promote the recruitment of CCR9^+^CD4^+^ T cells into the small intestine [105], leading to intestinal immune damage and gastrointestinal symptoms (Figure 6). Additionally, in the early stage of SARS-CoV-2 infection, the expression of CD68 in the duodenum and rectum of rhesus monkeys was significantly increased, and then returned to normal [76]. Increased CD68 expression was mainly located in the duodenum, jejunum, ileum, and descending colon, consistent with the expression of inflammatory cytokines [76].

### 4.2. Immune Response in the Gastrointestinal Tract Affects Lung

The gut–lung axis plays a vital role in the control of SARS-CoV-2 infection. SARS-CoV-2 infects the endothelial cells of blood vessels, and viral particles subsequently infiltrate the bloodstream and circulate throughout the body [106]. Inflammatory cytokines can be detected in the lung during the early stage of gastric infection of rhesus monkeys by SARS-CoV-2, including GM-CSF, IL1β, IL1rα, IL5, IL6, IL12, IL13, IL15, IL17A, IL18, MIP-1α, sCD40L, TGFα, TNF-α, and VEGF [76]. In the late stage of gastric infection with SARS-CoV-2, anti-inflammatory or protective cytokines, including G-CSF, interferon-γ, IL2, IL4, IL10, and MIP-1α, were found to increase in the lung, while inflammatory cytokines such as IL1β, IL1rα, IL5, IL6, IL8, IL15, and IL17A decreased [76]. Thus, cytokines can also enter the lungs through the bloodstream when the intestine is inflamed, thus affecting pulmonary immune responses and inflammation [107].

The microbiome has profound implications for human health and plays a major role in immunity. An increase in circulating pro-inflammatory cytokines also results in changes in the composition of the gut microbiome, leading to increased intestinal permeability, which in turn leads to the translocation of pathogens and toxins, increasing disease severity and multiple organ failure. A dysregulated gut environment combined with epithelial inflammation, in turn, increases ACE2 expression in the gut, and thus pro-inflammatory conditions within the gut microbiome improve the conditions favorable for SARS-CoV-2 infection [108]. There is a positive feedback loop between cytokines and inflammation, which worsens the prognosis of patients with COVID-19.

### 4.3. The Mechanism Pathogenesis of COVID-19-Associated Gastrointestinal Manifestation

SARS-CoV-2 infection disrupts the tight and adherent junctions of the endothelium and intestinal epithelium, which in turn may lead to leaky gut syndrome as well as local and systemic invasion of normal microbiota members, and immune activation [109]. By activating innate immune cells, IL-1β contributes to the development of a local inflammatory milieu and a systemic cytokine storm. RAS dysregulation may exacerbate ion imbalance and inflammation, potentially affecting cellular metabolic status, microbiota composition, and cell viability, leading to progressive bowel dysfunction and diarrhea [110].

The exact mechanism of nausea, vomiting, anorexia and abdominal pain associated with COVID-19 is unknown. Anorexia is often associated with other gastrointestinal symptoms such as vomiting, abdominal pain, and diarrhea. Abdominal pain combined with other gastrointestinal symptoms, such as anorexia, nausea, or vomiting. When ACE2-mediated SARS-CoV-2 directly invades gastrointestinal epithelial cells, if the immune system cannot defeat the infection, SARS-CoV-2 actively replicates in large numbers, resulting in reduced ACE2 levels and host cell destruction [74,111]. Gastrointestinal function is subsequently disrupted and inflammation is accelerated, causing nausea and vomiting [112]. Acute inflammation increases cytokine load, such as IL-2, IL-7, and TNF, which contribute to the cytokine storm seen in COVID-19. Any viral illness that is a prodrome can cause transient abdominal cramps and discomfort. After entering the gastrointestinal tract, SARS-CoV-2 can exert its cytopathic/inflammatory changes, resulting in visceral pain [113]. Furthermore, a recent study showed that CoV-2 infection of non-neuronal cell types caused anosmia and associated odor perception impairment in COVID-19 patients [114]. An altered sense of taste (dysgeusia) in these patients can further exacerbate anorexia [113,115].

## 5. Immunization and Prevention via the Gastrointestinal

Cellular and humoral immunity, mediated by T cells and B cells, plays a key role in COVID-19 [116,117,118]. B cell-derived antibodies to the spike protein and its receptor-binding domain prevent viral binding to epithelial cells [116,117]. Additionally, expansion of T follicular helper cells shows a mature humoral immune response that protects memory B cells from possible reinfection [116]. Current commercial vaccines are parenterally administered and primarily target the viral spike protein, a surface protein that undergoes significant antigenic drift. Consequently, adequate protection remains questionable.

### 5.1. Oral Vaccine Candidates

Current COVID-19 vaccines are designed to be administered by the parenteral intramuscular route and produce high titers of systemic neutralizing antibodies in response to systemic viral infection [119]. There are concerns about the persistence and effectiveness of mucosal immune responses following vaccination. High expression of the SARS-CoV-2 receptor ACE2 was observed in the ileum and colon of enterocytes of the digestive system [120]. While ACE2 was highly concentrated in the oronasal epithelium and the alveoli was lowest [121], it is speculated that the virus is in mucosal sites (oral/nasal) compared to more in-depth areas [122]. Oral vaccines have been successfully used for intestinal and respiratory infections and can effectively induce and activate the common mucosal immune system [119].

Oral vaccines induce strong antigen-specific IgG responses, mucosal IgA responses, and Th1/Th17 responses [123,124,125], thereby reducing or preventing viral infection and replication in the respiratory and intestinal mucosa. The potency and extent of oral vaccine-induced protective immunity can be assessed by monitoring the presence of the bacteria in feces and determining the level of protective antibodies present in the serum [126]. Furthermore, oral vaccines are cost-effective, easy to administer, easy to store, and widely accepted as biofriendly [127,128]. The development and use of oral vaccines against COVID-19 may also achieve broad immune protection in people in remote or underdeveloped countries [129]. Nonetheless, only a few SARS-CoV-2 vaccine candidates have been administered via the mucosal route.

For example, an oral multi-antigen SARS-CoV-2 vaccine, consisting of the receptor-binding domain of the viral spike protein, two domains of the viral nucleocapsid protein, and heat-stable enterotoxin B, can induce humoral, cellular, and mucosal immune responses, and provide immune protection [130]. In addition, the full-length receptor-binding domain of the SARS-CoV-2 spike protein is expressed on the surface of *S. cerevisiae*, and oral administration of this recombinant yeast induces significant humoral and mucosal responses and robust cellular immune response in mice [131]. Additionally, retrovirus-like particles expressing the SARS-CoV-2 spike and membrane proteins fused to a variable surface protein, modified with the intestinal parasite *Giardia*, elicited strong cellular and antibody immune responses and complete protection against SARS-CoV-2 in mice and hamsters after oral administration [132].

### 5.2. Maintain Intestinal Microbiota Homeostasis

There is a lot of evidence that probiotics can play a significant role in strengthening and regulating the immune system against disease [133,134,135]. Such *Lactobacillus* can act as an antiviral, leading to a symbiotic state in the gut microbiota, which can act as an anti-inflammatory and prevent superinfection [136]. Altered gut microbial composition, characterized by reduced commensal species and increased opportunistic pathogens, has been observed in patients with COVID-19 [137]. When the microbial flora is dysregulated, it is not only associated with intestinal barrier dysfunction, gastrointestinal diseases such as inflammatory bowel disease and colorectal cancer, but also with SARS-CoV-2 infection [138]. For example, SARS-CoV-2 infection has been associated with altered gut microbial communities in patients with elevated *Granulicatella* spp. and *Rothia mucilaginosa* found in the oral and gut microbiome [139]. Moreover, the circulating lipopolysaccharide-binding protein levels are elevated in critically ill patients and are associated with circulating inflammatory biomarkers and immune cells [137].

A healthy gut microbiota can control lung infections caused by SARS-CoV-2 by producing large immune cells, and dietary probiotics and prebiotics modulate the gut microbial environment. They may help maintain gut microbiota homeostasis and affect SARS-CoV-2 infection [140]. Recent research reports indicate that COVID-19 patients in the probiotic group experienced the resolution of diarrhea and the resolution of other symptoms; in addition, the estimated risk of respiratory failure, intensive care unit hospitalization, and mortality were significantly lower in the probiotic group [141]. Additionally, multiple clinical trials are underway to evaluate the effects of using probiotics and gut modifiers on the microbiome on COVID-19 [142]. Thus, alleviating gut symptoms and altering or modifying the gut microbial composition and their metabolites may also be a possible beneficial adjunctive therapy for COVID-19 [143,144].

## 6. Conclusions

Many asymptomatic carriers of COVID-19, so-called silent, presymptomatic or asymptomatic individuals, make this pandemic challenging to control. In a minority of patients with COVID-19, gastrointestinal symptoms such as diarrhea may be present with or precede the development of respiratory symptoms. The infection of the respiratory tract with SARS-CoV-2 affects the microbiome of the gastrointestinal tract; gastrointestinal infection affects the microorganisms and the immune response of the respiratory tract. However, gastrointestinal infections are often overlooked. Therefore, a focus on intestinal symptoms and the alteration or modification of gut microbes or their metabolites in response to COVID-19 may be a useful therapeutic option. In addition, the development of oral SARS-CoV-2 vaccine candidates that induce humoral and mucosal immune responses will likely contribute to controlling the COVID-19 outbreak.

## Data Availability

No new data were created or analyzed in this study. Data sharing is not applicable to this article.

## References

[1] Coronaviridae Study Group of the International Committee on Taxonomy of Viruses (2020). The species Severe acute respiratory syndrome-related coronavirus: Classifying 2019-nCoV and naming it SARS-CoV-2. Nat. Microbiol..

[2] WHO Coronavirus Disease (COVID-19) Situation Reports. https://www.who.int/emergencies/diseases/novel-coronavirus-2019/situation-reports.

[3] WHO (2005). Statement on the Second Meeting of the International Health Regulations Emergency Committee Regarding the Outbreak of Novel Coronavirus (2019-nCoV). https://www.who.int/news-room/detail/30-01-2020-statement-on-the-second-meeting-of-the-international-health-regulations-.

[4] Hu B., Guo H., Zhou P., Shi Z.L. (2021). Characteristics of SARS-CoV-2 and COVID-19. Nat. Rev. Microbiol..

[5] National Center for Immunization and Respiratory Diseases (NCIRD), Division of Viral Diseases SARS-CoV-2 Variant Classifications and Definitions. Last Updated 26 April 2022. https://www.cdc.gov/coronavirus/2019-ncov/variants/variant-classifications.html.

[6] Gao S.J., Guo H., Luo G. (2022). Omicron variant (B.1.1.529) of SARS-CoV-2, a global urgent public health alert!. J. Med. Virol..

[7] Parasa S., Desai M., Thoguluva Chandrasekar V., Patel H.K., Kennedy K.F., Roesch T., Spadaccini M., Colombo M., Gabbiadini R., Artifon E.L.A. (2020). Prevalence of Gastrointestinal Symptoms and Fecal Viral Shedding in Patients With Coronavirus Disease 2019: A Systematic Review and Meta-analysis. JAMA Netw. Open.

[8] Holshue M.L., DeBolt C., Lindquist S., Lofy K.H., Wiesman J., Bruce H., Spitters C., Ericson K., Wilkerson S., Tural A. (2020). First Case of 2019 Novel Coronavirus in the United States. N. Engl. J. Med..

[9] Cheung K.S., Hung I.F.N., Chan P.P.Y., Lung K.C., Tso E., Liu R., Ng Y.Y., Chu M.Y., Chung T.W.H., Tam A.R. (2020). Gastrointestinal Manifestations of SARS-CoV-2 Infection and Virus Load in Fecal Samples From a Hong Kong Cohort: Systematic Review and Meta-analysis. Gastroenterology.

[10] Ye Q., Wang B., Zhang T., Xu J., Shang S. (2020). The mechanism and treatment of gastrointestinal symptoms in patients with COVID-19. Am. J. Physiol. Gastrointest. Liver Physiol..

[11] Marasco G., Maida M., Morreale G.C., Licata M., Renzulli M., Cremon C., Stanghellini V., Barbara G. (2021). Gastrointestinal Bleeding in COVID-19 Patients: A Systematic Review with Meta-Analysis. Can. J. Gastroenterol. Hepatol..

[12] Shehab M., Alrashed F., Shuaibi S., Alajmi D., Barkun A. (2021). Gastroenterological and hepatic manifestations of patients with COVID-19, prevalence, mortality by country, and intensive care admission rate: Systematic review and meta-analysis. BMJ Open Gastroenterol..

[13] Pan L., Mu M., Yang P., Sun Y., Wang R., Yan J., Li P., Hu B., Wang J., Hu C. (2020). Clinical Characteristics of COVID-19 Patients With Digestive Symptoms in Hubei, China: A Descriptive, Cross-Sectional, Multicenter Study. Am. J. Gastroenterol..

[14] Guan W.J., Ni Z.Y., Hu Y., Liang W.H., Ou C.Q., He J.X., Liu L., Shan H., Lei C.L., Hui D.S.C. (2020). Clinical Characteristics of Coronavirus Disease 2019 in China. N. Engl. J. Med..

[15] Redd W.D., Zhou J.C., Hathorn K.E., McCarty T.R., Bazarbashi A.N., Thompson C.C., Shen L., Chan W.W. (2020). Prevalence and Characteristics of Gastrointestinal Symptoms in Patients With Severe Acute Respiratory Syndrome Coronavirus 2 Infection in the United States: A Multicenter Cohort Study. Gastroenterology.

[16] Lin L., Jiang X., Zhang Z., Huang S., Zhang Z., Fang Z., Gu Z., Gao L., Shi H., Mai L. (2020). Gastrointestinal symptoms of 95 cases with SARS-CoV-2 infection. Gut.

[17] Remes-Troche J.M., Ramos-de-la-Medina A., Manriquez-Reyes M., Martinez-Perez-Maldonado L., Lara E.L., Solis-Gonzalez M.A. (2020). Initial Gastrointestinal Manifestations in Patients With Severe Acute Respiratory Syndrome Coronavirus 2 Infection in 112 Patients From Veracruz in Southeastern Mexico. Gastroenterology.

[18] Boraschi P., Giugliano L., Mercogliano G., Donati F., Romano S., Neri E. (2021). Abdominal and gastrointestinal manifestations in COVID-19 patients: Is imaging useful?. World J. Gastroenterol..

[19] Zarifian A., Zamiri Bidary M., Arekhi S., Rafiee M., Gholamalizadeh H., Amiriani A., Ghaderi M.S., Khadem-Rezaiyan M., Amini M., Ganji A. (2021). Gastrointestinal and hepatic abnormalities in patients with confirmed COVID-19: A systematic review and meta-analysis. J. Med. Virol..

[20] Zeng W., Qi K., Ye M., Zheng L., Liu X., Hu S., Zhang W., Tang W., Xu J., Yu D. (2022). Gastrointestinal symptoms are associated with severity of coronavirus disease 2019: A systematic review and meta-analysis. Eur. J. Gastroenterol. Hepatol..

[21] Mao R., Qiu Y., He J.S., Tan J.Y., Li X.H., Liang J., Shen J., Zhu L.R., Chen Y., Iacucci M. (2020). Manifestations and prognosis of gastrointestinal and liver involvement in patients with COVID-19: A systematic review and meta-analysis. Lancet Gastroenterol. Hepatol..

[22] Wang J., Yuan X. (2021). Digestive system symptoms and function in children with COVID-19: A meta-analysis. Medicine.

[23] Bolia R., Dhanesh Goel A., Badkur M., Jain V. (2021). Gastrointestinal Manifestations of Pediatric Coronavirus Disease and Their Relationship with a Severe Clinical Course: A Systematic Review and Meta-analysis. J. Trop. Pediatr..

[24] Das S., Jayaratne R., Barrett K.E. (2018). The Role of Ion Transporters in the Pathophysiology of Infectious Diarrhea. Cell Mol. Gastroenterol. Hepatol..

[25] Sueyoshi R., Ignatoski K.M., Daignault S., Okawada M., Teitelbaum D.H. (2013). Angiotensin converting enzyme-inhibitor reduces colitis severity in an IL-10 knockout model. Dig. Dis. Sci..

[26] Kariyawasam J.C., Jayarajah U., Riza R., Abeysuriya V., Seneviratne S.L. (2021). Gastrointestinal manifestations in COVID-19. Trans. R. Soc. Trop. Med. Hyg..

[27] Luo S., Zhang X., Xu H. (2020). Don’t Overlook Digestive Symptoms in Patients With 2019 Novel Coronavirus Disease (COVID-19). Clin. Gastroenterol. Hepatol..

[28] Jin X., Lian J.S., Hu J.H., Gao J., Zheng L., Zhang Y.M., Hao S.R., Jia H.Y., Cai H., Zhang X.L. (2020). Epidemiological, clinical and virological characteristics of 74 cases of coronavirus-infected disease 2019 (COVID-19) with gastrointestinal symptoms. Gut.

[29] Lee I.C., Huo T.I., Huang Y.H. (2020). Gastrointestinal and liver manifestations in patients with COVID-19. J. Chin. Med. Assoc..

[30] Wang H., Qiu P., Liu J., Wang F., Zhao Q. (2020). The liver injury and gastrointestinal symptoms in patients with Coronavirus Disease 19: A systematic review and meta-analysis. Clin. Res. Hepatol. Gastroenterol..

[31] Tian Y., Rong L., Nian W., He Y. (2020). Review article: Gastrointestinal features in COVID-19 and the possibility of faecal transmission. Aliment. Pharmacol. Ther..

[32] El Moheb M., Naar L., Christensen M.A., Kapoen C., Maurer L.R., Farhat M., Kaafarani H.M.A. (2020). Gastrointestinal Complications in Critically Ill Patients With and Without COVID-19. JAMA.

[33] Nobel Y.R., Phipps M., Zucker J., Lebwohl B., Wang T.C., Sobieszczyk M.E., Freedberg D.E. (2020). Gastrointestinal Symptoms and Coronavirus Disease 2019: A Case-Control Study From the United States. Gastroenterology.

[34] Aghemo A., Piovani D., Parigi T.L., Brunetta E., Pugliese N., Vespa E., Omodei P.D., Preatoni P., Lleo A., Repici A. (2020). COVID-19 Digestive System Involvement and Clinical Outcomes in a Large Academic Hospital in Milan, Italy. Clin. Gastroenterol. Hepatol..

[35] Livanos A.E., Jha D., Cossarini F., Gonzalez-Reiche A.S., Tokuyama M., Aydillo T., Parigi T.L., Ladinsky M.S., Ramos I., Dunleavy K. (2021). Intestinal Host Response to SARS-CoV-2 Infection and COVID-19 Outcomes in Patients with Gastrointestinal Symptoms. Gastroenterology.

[36] Klok F.A., Kruip M., van der Meer N.J.M., Arbous M.S., Gommers D., Kant K.M., Kaptein F.H.J., van Paassen J., Stals M.A.M., Huisman M.V. (2020). Incidence of thrombotic complications in critically ill ICU patients with COVID-19. Thromb. Res..

[37] Connors J.M., Levy J.H. (2020). COVID-19 and its implications for thrombosis and anticoagulation. Blood.

[38] Wichmann D., Sperhake J.P., Lutgehetmann M., Steurer S., Edler C., Heinemann A., Heinrich F., Mushumba H., Kniep I., Schroder A.S. (2020). Autopsy Findings and Venous Thromboembolism in Patients With COVID-19: A Prospective Cohort Study. Ann. Intern. Med..

[39] Salehi S., Gholamrezanezhad A. (2020). Reply to “Segmental Pulmonary Vascular Changes in COVID-19 Pneumonia”. AJR Am. J. Roentgenol..

[40] Salehi S., Abedi A., Gholamrezanezhad A. (2020). Reply to “Vascular Changes Detected With Thoracic CT in Coronavirus Disease (COVID-19) Might Be Significant Determinants for Accurate Diagnosis and Optimal Patient Management”. AJR Am. J. Roentgenol..

[41] Eslambolchi A., Aghaghazvini L., Gholamrezanezhad A., Kavosi H., Radmard A.R. (2021). Coronavirus disease 2019 (COVID-19) in patients with systemic autoimmune diseases or vasculitis: Radiologic presentation. J. Thromb. Thrombolysis.

[42] Kooraki S., Hosseiny M., Myers L., Gholamrezanezhad A. (2020). Coronavirus (COVID-19) Outbreak: What the Department of Radiology Should Know. J. Am. Coll. Radiol..

[43] Keshavarz P., Rafiee F., Kavandi H., Goudarzi S., Heidari F., Gholamrezanezhad A. (2021). Ischemic gastrointestinal complications of COVID-19: A systematic review on imaging presentation. Clin. Imaging.

[44] Subissi L., Posthuma C.C., Collet A., Zevenhoven-Dobbe J.C., Gorbalenya A.E., Decroly E., Snijder E.J., Canard B., Imbert I. (2014). One severe acute respiratory syndrome coronavirus protein complex integrates processive RNA polymerase and exonuclease activities. Proc. Natl. Acad. Sci. USA.

[45] Rota P.A., Oberste M.S., Monroe S.S., Nix W.A., Campagnoli R., Icenogle J.P., Penaranda S., Bankamp B., Maher K., Chen M.H. (2003). Characterization of a novel coronavirus associated with severe acute respiratory syndrome. Science.

[46] V’Kovski P., Kratzel A., Steiner S., Stalder H., Thiel V. (2021). Coronavirus biology and replication: Implications for SARS-CoV-2. Nat. Rev. Microbiol..

[47] Brian D.A., Baric R.S. (2005). Coronavirus genome structure and replication. Curr. Top Microbiol. Immunol..

[48] Henry B.M., de Oliveira M.H.S., Benoit S., Plebani M., Lippi G. (2020). Hematologic, biochemical and immune biomarker abnormalities associated with severe illness and mortality in coronavirus disease 2019 (COVID-19): A meta-analysis. Clin. Chem. Lab. Med..

[49] Choudhry N., Zhao X., Xu D., Zanin M., Chen W., Yang Z., Chen J. (2020). Chinese Therapeutic Strategy for Fighting COVID-19 and Potential Small-Molecule Inhibitors against Severe Acute Respiratory Syndrome Coronavirus 2 (SARS-CoV-2). J. Med. Chem..

[50] Jackson C.B., Farzan M., Chen B., Choe H. (2022). Mechanisms of SARS-CoV-2 entry into cells. Nat. Rev. Mol. Cell Biol..

[51] Yan R., Zhang Y., Li Y., Xia L., Guo Y., Zhou Q. (2020). Structural basis for the recognition of SARS-CoV-2 by full-length human ACE2. Science.

[52] Wrapp D., Wang N., Corbett K.S., Goldsmith J.A., Hsieh C.L., Abiona O., Graham B.S., McLellan J.S. (2020). Cryo-EM structure of the 2019-nCoV spike in the prefusion conformation. Science.

[53] Parmar M.S. (2021). TMPRSS2: An Equally Important Protease as ACE2 in the Pathogenicity of SARS-CoV-2 Infection. Mayo Clin. Proc..

[54] Wu L., Zhou L., Mo M., Liu T., Wu C., Gong C., Lu K., Gong L., Zhu W., Xu Z. (2022). SARS-CoV-2 Omicron RBD shows weaker binding affinity than the currently dominant Delta variant to human ACE2. Signal Transduct. Target. Ther..

[55] Zou X., Chen K., Zou J., Han P., Hao J., Han Z. (2020). Single-cell RNA-seq data analysis on the receptor ACE2 expression reveals the potential risk of different human organs vulnerable to 2019-nCoV infection. Front. Med..

[56] Zhao Y., Zhao Z., Wang Y., Zhou Y., Ma Y., Zuo W. (2020). Single-Cell RNA Expression Profiling of ACE2, the Receptor of SARS-CoV-2. Am. J. Respir. Crit. Care Med..

[57] Xu H., Zhong L., Deng J., Peng J., Dan H., Zeng X., Li T., Chen Q. (2020). High expression of ACE2 receptor of 2019-nCoV on the epithelial cells of oral mucosa. Int. J. Oral Sci..

[58] Liang W., Feng Z., Rao S., Xiao C., Xue X., Lin Z., Zhang Q., Qi W. (2020). Diarrhoea may be underestimated: A missing link in 2019 novel coronavirus. Gut.

[59] He J., Tao H., Yan Y., Huang S.Y., Xiao Y. (2020). Molecular Mechanism of Evolution and Human Infection with SARS-CoV-2. Viruses.

[60] Hoffmann M., Kleine-Weber H., Schroeder S., Kruger N., Herrler T., Erichsen S., Schiergens T.S., Herrler G., Wu N.H., Nitsche A. (2020). SARS-CoV-2 Cell Entry Depends on ACE2 and TMPRSS2 and Is Blocked by a Clinically Proven Protease Inhibitor. Cell.

[61] Lamers M.M., Beumer J., van der Vaart J., Knoops K., Puschhof J., Breugem T.I., Ravelli R.B.G., Paul van Schayck J., Mykytyn A.Z., Duimel H.Q. (2020). SARS-CoV-2 productively infects human gut enterocytes. Science.

[62] Sungnak W., Huang N., Becavin C., Berg M., Queen R., Litvinukova M., Talavera-Lopez C., Maatz H., Reichart D., Sampaziotis F. (2020). SARS-CoV-2 entry factors are highly expressed in nasal epithelial cells together with innate immune genes. Nat. Med..

[63] Berni Canani R., Comegna M., Paparo L., Cernera G., Bruno C., Strisciuglio C., Zollo I., Gravina A.G., Miele E., Cantone E. (2021). Age-Related Differences in the Expression of Most Relevant Mediators of SARS-CoV-2 Infection in Human Respiratory and Gastrointestinal Tract. Front. Pediatr..

[64] Gu J., Han B., Wang J. (2020). COVID-19: Gastrointestinal Manifestations and Potential Fecal-Oral Transmission. Gastroenterology.

[65] Li W., Moore M.J., Vasilieva N., Sui J., Wong S.K., Berne M.A., Somasundaran M., Sullivan J.L., Luzuriaga K., Greenough T.C. (2003). Angiotensin-converting enzyme 2 is a functional receptor for the SARS coronavirus. Nature.

[66] Hashimoto T., Perlot T., Rehman A., Trichereau J., Ishiguro H., Paolino M., Sigl V., Hanada T., Hanada R., Lipinski S. (2012). ACE2 links amino acid malnutrition to microbial ecology and intestinal inflammation. Nature.

[67] Kuba K., Imai Y., Rao S., Gao H., Guo F., Guan B., Huan Y., Yang P., Zhang Y., Deng W. (2005). A crucial role of angiotensin converting enzyme 2 (ACE2) in SARS coronavirus-induced lung injury. Nat. Med..

[68] Cao Y., Li L., Feng Z., Wan S., Huang P., Sun X., Wen F., Huang X., Ning G., Wang W. (2020). Comparative genetic analysis of the novel coronavirus (2019-nCoV/SARS-CoV-2) receptor ACE2 in different populations. Cell Discov..

[69] Irham L.M., Chou W.H., Calkins M.J., Adikusuma W., Hsieh S.L., Chang W.C. (2020). Genetic variants that influence SARS-CoV-2 receptor TMPRSS2 expression among population cohorts from multiple continents. Biochem. Biophys. Res. Commun..

[70] Asselta R., Paraboschi E.M., Mantovani A., Duga S. (2020). ACE2 and TMPRSS2 variants and expression as candidates to sex and country differences in COVID-19 severity in Italy. Aging.

[71] Spencer L., Olawuni B., Singh P. (2022). Gut Virome: Role and Distribution in Health and Gastrointestinal Diseases. Front. Cell Infect. Microbiol..

[72] Yeo C., Kaushal S., Yeo D. (2020). Enteric involvement of coronaviruses: Is faecal-oral transmission of SARS-CoV-2 possible?. Lancet Gastroenterol. Hepatol..

[73] Wang W., Xu Y., Gao R., Lu R., Han K., Wu G., Tan W. (2020). Detection of SARS-CoV-2 in Different Types of Clinical Specimens. JAMA.

[74] Xiao F., Tang M., Zheng X., Liu Y., Li X., Shan H. (2020). Evidence for Gastrointestinal Infection of SARS-CoV-2. Gastroenterology.

[75] Li L.Y., Wu W., Chen S., Gu J.W., Li X.L., Song H.J., Du F., Wang G., Zhong C.Q., Wang X.Y. (2020). Digestive system involvement of novel coronavirus infection: Prevention and control infection from a gastroenterology perspective. J. Dig. Dis..

[76] Jiao L., Li H., Xu J., Yang M., Ma C., Li J., Zhao S., Wang H., Yang Y., Yu W. (2021). The Gastrointestinal Tract Is an Alternative Route for SARS-CoV-2 Infection in a Nonhuman Primate Model. Gastroenterology.

[77] Wu Y., Guo C., Tang L., Hong Z., Zhou J., Dong X., Yin H., Xiao Q., Tang Y., Qu X. (2020). Prolonged presence of SARS-CoV-2 viral RNA in faecal samples. Lancet Gastroenterol. Hepatol..

[78] Dieterich W., Schink M., Zopf Y. (2018). Microbiota in the Gastrointestinal Tract. Med. Sci..

[79] Gilbert J.A., Lynch S.V. (2019). Community ecology as a framework for human microbiome research. Nat. Med..

[80] Human Microbiome Project C. (2012). Structure, function and diversity of the healthy human microbiome. Nature.

[81] Golonka R.M., Saha P., Yeoh B.S., Chattopadhyay S., Gewirtz A.T., Joe B., Vijay-Kumar M. (2020). Harnessing innate immunity to eliminate SARS-CoV-2 and ameliorate COVID-19 disease. Physiol. Genom..

[82] Xiao F., Sun J., Xu Y., Li F., Huang X., Li H., Zhao J., Huang J., Zhao J. (2020). Infectious SARS-CoV-2 in Feces of Patient with Severe COVID-19. Emerg. Infect. Dis..

[83] Mazza S., Sorce A., Peyvandi F., Vecchi M., Caprioli F. (2020). A fatal case of COVID-19 pneumonia occurring in a patient with severe acute ulcerative colitis. Gut.

[84] Farsi Y., Tahvildari A., Arbabi M., Vazife F., Sechi L.A., Shahidi Bonjar A.H., Jamshidi P., Nasiri M.J., Mirsaeidi M. (2022). Diagnostic, Prognostic, and Therapeutic Roles of Gut Microbiota in COVID-19: A Comprehensive Systematic Review. Front. Cell Infect. Microbiol..

[85] Budden K.F., Gellatly S.L., Wood D.L., Cooper M.A., Morrison M., Hugenholtz P., Hansbro P.M. (2017). Emerging pathogenic links between microbiota and the gut-lung axis. Nat. Rev. Microbiol..

[86] Reinold J., Farahpour F., Schoerding A.K., Fehring C., Dolff S., Konik M., Korth J., van Baal L., Buer J., Witzke O. (2022). The Fungal Gut Microbiome Exhibits Reduced Diversity and Increased Relative Abundance of Ascomycota in Severe COVID-19 Illness and Distinct Interconnected Communities in SARS-CoV-2 Positive Patients. Front. Cell Infect. Microbiol..

[87] Hazan S., Stollman N., Bozkurt H.S., Dave S., Papoutsis A.J., Daniels J., Barrows B.D., Quigley E.M., Borody T.J. (2022). Lost microbes of COVID-19: Bifidobacterium, Faecalibacterium depletion and decreased microbiome diversity associated with SARS-CoV-2 infection severity. BMJ Open Gastroenterol..

[88] Sankova M.V., Nikolenko V.N., Oganesyan M.V., Bakhmet A.A., Gavryushova L.V., Sankov S.V., Sinelnikov M.Y. (2022). Current drug targets for gut microbiota biocorrection during the SARS-CoV-2 pandemic: A systematic review. Curr. Drug Targets.

[89] Cheung C.Y., Poon L.L., Ng I.H., Luk W., Sia S.F., Wu M.H., Chan K.H., Yuen K.Y., Gordon S., Guan Y. (2005). Cytokine responses in severe acute respiratory syndrome coronavirus-infected macrophages in vitro: Possible relevance to pathogenesis. J. Virol..

[90] Lau S.K.P., Lau C.C.Y., Chan K.H., Li C.P.Y., Chen H., Jin D.Y., Chan J.F.W., Woo P.C.Y., Yuen K.Y. (2013). Delayed induction of proinflammatory cytokines and suppression of innate antiviral response by the novel Middle East respiratory syndrome coronavirus: Implications for pathogenesis and treatment. J. Gen. Virol..

[91] Law H.K., Cheung C.Y., Ng H.Y., Sia S.F., Chan Y.O., Luk W., Nicholls J.M., Peiris J.S., Lau Y.L. (2005). Chemokine up-regulation in SARS-coronavirus-infected, monocyte-derived human dendritic cells. Blood.

[92] Mathew D., Giles J.R., Baxter A.E., Oldridge D.A., Greenplate A.R., Wu J.E., Alanio C., Kuri-Cervantes L., Pampena M.B., D’Andrea K. (2020). Deep immune profiling of COVID-19 patients reveals distinct immunotypes with therapeutic implications. Science.

[93] Wilk A.J., Rustagi A., Zhao N.Q., Roque J., Martinez-Colon G.J., McKechnie J.L., Ivison G.T., Ranganath T., Vergara R., Hollis T. (2020). A single-cell atlas of the peripheral immune response in patients with severe COVID-19. Nat. Med..

[94] He L., Ding Y., Zhang Q., Che X., He Y., Shen H., Wang H., Li Z., Zhao L., Geng J. (2006). Expression of elevated levels of pro-inflammatory cytokines in SARS-CoV-infected ACE2+ cells in SARS patients: Relation to the acute lung injury and pathogenesis of SARS. J. Pathol..

[95] Moore J.B., June C.H. (2020). Cytokine release syndrome in severe COVID-19. Science.

[96] Group R.C., Horby P., Lim W.S., Emberson J.R., Mafham M., Bell J.L., Linsell L., Staplin N., Brightling C., Ustianowski A. (2021). Dexamethasone in Hospitalized Patients with Covid-19. N. Engl. J. Med..

[97] Huang C., Wang Y., Li X., Ren L., Zhao J., Hu Y., Zhang L., Fan G., Xu J., Gu X. (2020). Clinical features of patients infected with 2019 novel coronavirus in Wuhan, China. Lancet.

[98] Zhu Z., Cai T., Fan L., Lou K., Hua X., Huang Z., Gao G. (2020). Clinical value of immune-inflammatory parameters to assess the severity of coronavirus disease 2019. Int. J. Infect. Dis..

[99] Del Valle D.M., Kim-Schulze S., Huang H.H., Beckmann N.D., Nirenberg S., Wang B., Lavin Y., Swartz T.H., Madduri D., Stock A. (2020). An inflammatory cytokine signature predicts COVID-19 severity and survival. Nat. Med..

[100] Wan Y., Shang J., Graham R., Baric R.S., Li F. (2020). Receptor Recognition by the Novel Coronavirus from Wuhan: An Analysis Based on Decade-Long Structural Studies of SARS Coronavirus. J. Virol..

[101] Jeyanathan M., Afkhami S., Smaill F., Miller M.S., Lichty B.D., Xing Z. (2020). Immunological considerations for COVID-19 vaccine strategies. Nat. Rev. Immunol..

[102] Sterlin D., Mathian A., Miyara M., Mohr A., Anna F., Claer L., Quentric P., Fadlallah J., Devilliers H., Ghillani P. (2021). IgA dominates the early neutralizing antibody response to SARS-CoV-2. Sci. Transl. Med..

[103] Wang J., Li F., Wei H., Lian Z.X., Sun R., Tian Z. (2014). Respiratory influenza virus infection induces intestinal immune injury via microbiota-mediated Th17 cell-dependent inflammation. J. Exp. Med..

[104] Papadakis K.A., Prehn J., Nelson V., Cheng L., Binder S.W., Ponath P.D., Andrew D.P., Targan S.R. (2000). The role of thymus-expressed chemokine and its receptor CCR9 on lymphocytes in the regional specialization of the mucosal immune system. J. Immunol..

[105] Stenstad H., Ericsson A., Johansson-Lindbom B., Svensson M., Marsal J., Mack M., Picarella D., Soler D., Marquez G., Briskin M. (2006). Gut-associated lymphoid tissue-primed CD4^+^ T cells display CCR9-dependent and -independent homing to the small intestine. Blood.

[106] Varga Z., Flammer A.J., Steiger P., Haberecker M., Andermatt R., Zinkernagel A.S., Mehra M.R., Schuepbach R.A., Ruschitzka F., Moch H. (2020). Endothelial cell infection and endotheliitis in COVID-19. Lancet.

[107] Zhang D., Li S., Wang N., Tan H.Y., Zhang Z., Feng Y. (2020). The Cross-Talk Between Gut Microbiota and Lungs in Common Lung Diseases. Front. Microbiol..

[108] Chan J.F., Kok K.H., Zhu Z., Chu H., To K.K., Yuan S., Yuen K.Y. (2020). Genomic characterization of the 2019 novel human-pathogenic coronavirus isolated from a patient with atypical pneumonia after visiting Wuhan. Emerg. Microbes. Infect..

[109] Megyeri K., Dernovics A., Al-Luhaibi Z.I.I., Rosztoczy A. (2021). COVID-19-associated diarrhea. World J. Gastroenterol..

[110] Delorme-Axford E., Klionsky D.J. (2020). Highlights in the fight against COVID-19: Does autophagy play a role in SARS-CoV-2 infection?. Autophagy.

[111] Xiao L., Sakagami H., Miwa N. (2020). ACE2: The key Molecule for Understanding the Pathophysiology of Severe and Critical Conditions of COVID-19: Demon or Angel?. Viruses.

[112] Zhang T., Liu D., Tian D., Xia L. (2021). The roles of nausea and vomiting in COVID-19: Did we miss something?. J. Microbiol. Immunol. Infect.

[113] Perisetti A., Goyal H., Gajendran M., Boregowda U., Mann R., Sharma N. (2020). Prevalence, Mechanisms, and Implications of Gastrointestinal Symptoms in COVID-19. Front. Med..

[114] Brann D.H., Tsukahara T., Weinreb C., Lipovsek M., Van den Berge K., Gong B., Chance R., Macaulay I.C., Chou H.J., Fletcher R.B. (2020). Non-neuronal expression of SARS-CoV-2 entry genes in the olfactory system suggests mechanisms underlying COVID-19-associated anosmia. Sci. Adv..

[115] Aziz M., Perisetti A., Lee-Smith W.M., Gajendran M., Bansal P., Goyal H. (2020). Taste Changes (Dysgeusia) in COVID-19: A Systematic Review and Meta-analysis. Gastroenterology.

[116] Cox R.J., Brokstad K.A. (2020). Not just antibodies: B cells and T cells mediate immunity to COVID-19. Nat. Rev. Immunol..

[117] Grifoni A., Weiskopf D., Ramirez S.I., Mateus J., Dan J.M., Moderbacher C.R., Rawlings S.A., Sutherland A., Premkumar L., Jadi R.S. (2020). Targets of T Cell Responses to SARS-CoV-2 Coronavirus in Humans with COVID-19 Disease and Unexposed Individuals. Cell.

[118] Sekine T., Perez-Potti A., Rivera-Ballesteros O., Stralin K., Gorin J.B., Olsson A., Llewellyn-Lacey S., Kamal H., Bogdanovic G., Muschiol S. (2020). Robust T Cell Immunity in Convalescent Individuals with Asymptomatic or Mild COVID-19. Cell.

[119] Ashraf M.U., Kim Y., Kumar S., Seo D., Ashraf M., Bae Y.S. (2021). COVID-19 Vaccines (Revisited) and Oral-Mucosal Vector System as a Potential Vaccine Platform. Vaccines.

[120] Zhang H., Kang Z., Gong H., Xu D., Wang J., Li Z., Li Z., Cui X., Xiao J., Zhan J. (2020). Digestive system is a potential route of COVID-19: An analysis of single-cell coexpression pattern of key proteins in viral entry process. Gut.

[121] Hou Y.J., Okuda K., Edwards C.E., Martinez D.R., Asakura T., Dinnon K.H., Kato T., Lee R.E., Yount B.L., Mascenik T.M. (2020). SARS-CoV-2 Reverse Genetics Reveals a Variable Infection Gradient in the Respiratory Tract. Cell.

[122] Sims A.C., Baric R.S., Yount B., Burkett S.E., Collins P.L., Pickles R.J. (2005). Severe acute respiratory syndrome coronavirus infection of human ciliated airway epithelia: Role of ciliated cells in viral spread in the conducting airways of the lungs. J. Virol.

[123] Liu M., Zhong Y., Chen J., Liu Y., Tang C., Wang X., Zhang Y., Wang P., Logan S.M., Chen W. (2020). Oral immunization of mice with a multivalent therapeutic subunit vaccine protects against Helicobacter pylori infection. Vaccine.

[124] Wang S., Geng N., Zhou D., Qu Y., Shi M., Xu Y., Liu K., Liu Y., Liu J. (2019). Oral Immunization of Chickens With Recombinant Lactobacillus plantarum Vaccine Against Early ALV-J Infection. Front. Immunol..

[125] Mustafa A.D., Kalyanasundram J., Sabidi S., Song A.A., Abdullah M., Abdul Rahim R., Yusoff K. (2018). Proof of concept in utilizing in-trans surface display system of Lactobacillus plantarum as mucosal tuberculosis vaccine via oral administration in mice. BMC Biotechnol..

[126] Baker P.J. (2022). Advantages of an Oral Vaccine to Control the COVID-19 Pandemic. Am. J. Med..

[127] Shakoor S., Rao A.Q., Shahid N., Yaqoob A., Samiullah T.R., Shakoor S., Latif A., Tabassum B., Khan M.A.U., Shahid A.A. (2019). Role of oral vaccines as an edible tool to prevent infectious diseases. Acta Virol..

[128] Savoie K. (2000). Edible vaccine success. Nat. Biotechnol..

[129] del Rio B., Dattwyler R.J., Aroso M., Neves V., Meirelles L., Seegers J.F., Gomes-Solecki M. (2008). Oral immunization with recombinant lactobacillus plantarum induces a protective immune response in mice with Lyme disease. Clin. Vaccine Immunol..

[130] Pitcovski J., Gruzdev N., Abzach A., Katz C., Ben-Adiva R., Brand-Shwartz M., Yadid I., Ratzon-Ashkenazi E., Emquies K., Israeli H. (2022). Oral subunit SARS-CoV-2 vaccine induces systemic neutralizing IgG, IgA and cellular immune responses and can boost neutralizing antibody responses primed by an injected vaccine. Vaccine.

[131] Gao T., Ren Y., Li S., Lu X., Lei H. (2021). Immune response induced by oral administration with a Saccharomyces cerevisiae-based SARS-CoV-2 vaccine in mice. Microb. Cell Fact..

[132] Bellier B., Saura A., Lujan L.A., Molina C.R., Lujan H.D., Klatzmann D. (2022). A Thermostable Oral SARS-CoV-2 Vaccine Induces Mucosal and Protective Immunity. Front. Immunol..

[133] Ballini A., Santacroce L., Cantore S., Bottalico L., Dipalma G., Topi S., Saini R., De Vito D., Inchingolo F. (2019). Probiotics Efficacy on Oxidative Stress Values in Inflammatory Bowel Disease: A Randomized Double-Blinded Placebo-Controlled Pilot Study. Endocr. Metab. Immune Disord. Drug Targets.

[134] Santacroce L., Charitos I.A., Bottalico L. (2019). A successful history: Probiotics and their potential as antimicrobials. Expert Rev. Anti Infect. Ther.

[135] Santacroce L., Inchingolo F., Topi S., Del Prete R., Di Cosola M., Charitos I.A., Montagnani M. (2021). Potential beneficial role of probiotics on the outcome of COVID-19 patients: An evolving perspective. Diabetes Metab. Syndr..

[136] Mu Q., Tavella V.J., Luo X.M. (2018). Role of Lactobacillus reuteri in Human Health and Diseases. Front. Microbiol..

[137] Sun Z., Song Z.G., Liu C., Tan S., Lin S., Zhu J., Dai F.H., Gao J., She J.L., Mei Z. (2022). Gut microbiome alterations and gut barrier dysfunction are associated with host immune homeostasis in COVID-19 patients. BMC Med..

[138] Zuo T., Zhang F., Lui G.C.Y., Yeoh Y.K., Li A.Y.L., Zhan H., Wan Y., Chung A.C.K., Cheung C.P., Chen N. (2020). Alterations in Gut Microbiota of Patients With COVID-19 During Time of Hospitalization. Gastroenterology.

[139] Wu Y., Cheng X., Jiang G., Tang H., Ming S., Tang L., Lu J., Guo C., Shan H., Huang X. (2021). Altered oral and gut microbiota and its association with SARS-CoV-2 viral load in COVID-19 patients during hospitalization. NPJ Biofilms Microbiomes.

[140] Rajput S., Paliwal D., Naithani M., Kothari A., Meena K., Rana S. (2021). COVID-19 and Gut Microbiota: A Potential Connection. Indian J. Clin. Biochem..

[141] d’Ettorre G., Ceccarelli G., Marazzato M., Campagna G., Pinacchio C., Alessandri F., Ruberto F., Rossi G., Celani L., Scagnolari C. (2020). Challenges in the Management of SARS-CoV2 Infection: The Role of Oral Bacteriotherapy as Complementary Therapeutic Strategy to Avoid the Progression of COVID-19. Front. Med..

[142] de Ponte M.C., Cardoso V.G., Goncalves G.L., Costa-Pessoa J.M., Oliveira-Souza M. (2021). Early type 1 diabetes aggravates renal ischemia/reperfusion-induced acute kidney injury. Sci. Rep..

[143] Villapol S. (2020). Gastrointestinal symptoms associated with COVID-19: Impact on the gut microbiome. Transl. Res..

[144] Chen J., Vitetta L. (2021). Modulation of Gut Microbiota for the Prevention and Treatment of COVID-19. J. Clin. Med..

